# Gunshot Wounds

**Published:** 1848-06

**Authors:** 


*Gunshot Wounds.—A Clinical Lecture by Professor Velpeau.*—Gunshot wounds may interest the limbs or the body; we will first study them in the extremities. Of the ebrasure of the surface of the limbs by shot we will say nothing, these wounds not presenting habitually any importance. They are said to be simple when the soft parts only have been injured; they are called compound when the bones, nerves, or vessels have suffered. Laceration of the nerves is a less severe complication than rupture of the vessels; and the latter has not so much gravity as fracture of the bones, unless, indeed, the divided arteries be of a large size. Fractures, complicated by ordinary wounds, have not by any means so formidable a character as those which are accompanied by gunshot wounds, on account of the destruction of tissues caused by the passage of the foreign body, the subsequent mortification, and the rupture of the bone into numerous fragments. We should also observe that the protracted suppuration which follows exposes the patient to the threefold peril—of purulent suffusion in the limb, absorption of pus, or fatal debility.

The wounds inflicted by firearms do not cause, in all parts of the body, fractures of equal gravity. The fracture of two bones in one part, the vicinity of a joint to the injured region, are most serious circumstances in the prognosis. A bone may be simply perforated by the bullet, or its surface grazed by the passage of shot, without complete fracture; in these cases the peril is not great. Fractures of the inferior extremity are far more severe than those of the upper; the most dangerous of all are those of the thigh: the form of the bone, and the nature of its tissue, cause its fractures to be comminuted in almost every instance; besides, the deep situation of the bone renders purulent suffusion of the limb an accident of easy production.

In the treatment of gunshot wounds of the limbs the question of utmost importance to the practitioner is that of immediate or secondary amputation.

In order to solve that question as far as it is in his power, in each particular case, he should recollect that these wounds present three periods. The first, which lasts from twenty-four to thirty-six hours, is characterized by stupor, and is the result of the sudden disturbance occasioned in the nervous system by the wound received whilst the subject is in a high state of excitement. The second period is marked by inflammatory reaction, and is analogous to the stage of elimination of burns. The portions of tissues which have been in contact with the bullet are disorganized by the violence of its passage, and must be removed by the efforts of nature. During the first three days of this second period, swelling and inflammation are observed; they soon cause an ichorous and thin fluid to be secreted, and finally the formation of true pus.

Amputation should, if necessary, be performed during the period of stupor, and not during the inflammatory stage. The task of the surgeon is often very embarrassing, and it is most difficult in some cases to come to a decision, the deceptive hope of preserving the limb often inducing the practitioner to defer amputation until it is too late to save even life. Fractures of the thigh are, of all, the most difficult, appearing often not very serious, and yet melancholy experience informs us that almost invariably they require amputation. Such is, at least, the opinion of Larrey, Perey, &c. When these wounds are in the neighbourhood of a joint, the operation is imperatively demanded.

Between the first and second periods of gunshot wounds, hemorrhage or gangrene may take place: if abundant loss of blood follows immediately the stage of stupor, the limb should be removed at once. We will say the same if mortification shows itself, and we do not admit that the surgeon should wait for the limitation of the gangrene. In none of our cases have we used chloroform; we believe that it might increase in a dangerous degree the stupor of the nervous system already produced by the wounds.—*Medical Times.*

